# Vitamin D Deficiency Meets Hill’s Criteria for Causation in SARS-CoV-2 Susceptibility, Complications, and Mortality: A Systematic Review

**DOI:** 10.3390/nu17030599

**Published:** 2025-02-06

**Authors:** Sunil J. Wimalawansa

**Affiliations:** Endocrinology and Human Nutrition, CardioMetabolic & Endocrine Institute, North Brunswick, NJ 08902, USA; suniljw@hotmail.com

**Keywords:** 25(OH)D, 1,25(OH)_2_D, association, epidemiology, causality, COVID-19, micronutrients, public health, viral infections

## Abstract

Clinical trials consistently demonstrate an inverse correlation between serum 25-hydroxyvitamin D [25(OH)D; calcifediol] levels and the risk of symptomatic SARS-CoV-2 disease, complications, and mortality. This systematic review (SR), guided by Bradford Hill’s causality criteria, analyzed 294 peer-reviewed manuscripts published between December 2019 and November 2024, focusing on plausibility, consistency, and biological gradient. Evidence confirms that cholecalciferol (D_3_) and calcifediol significantly reduce symptomatic disease, complications, hospitalizations, and mortality, with optimal effects above 50 ng/mL. While vitamin D requires 3–4 days to act, calcifediol shows effects within 24 h. Among 329 trials, only 11 (3%) showed no benefit due to flawed designs. At USD 2/patient, D_3_ supplementation is far cheaper than hospitalization costs and more effective than standard interventions. This SR establishes a strong inverse relationship between 25(OH)D levels and SARS-CoV-2 vulnerability, meeting Hill’s criteria. Vitamin D_3_ and calcifediol reduce infections, complications, hospitalizations, and deaths by ~50%, outperforming all patented, FDA-approved COVID-19 therapies. With over 300 trials confirming these findings, waiting for further studies is unnecessary before incorporating them into clinical protocols. Health agencies and scientific societies must recognize the significance of these results and incorporate D_3_ and calcifediol for prophylaxis and early treatment protocols of SARS-CoV-2 and similar viral infections. Promoting safe sun exposure and adequate vitamin D_3_ supplementation within communities to maintain 25(OH)D levels above 40 ng/mL (therapeutic range: 40–80 ng/mL) strengthens immune systems, reduces hospitalizations and deaths, and significantly lowers healthcare costs. When serum 25(OH)D levels exceed 70 ng/mL, taking vitamin K_2_ (100 µg/day or 800 µg/week) alongside vitamin D helps direct any excess calcium to bones. The recommended vitamin D dosage (approximately 70 IU/kg of body weight for a non-obese adult) to maintain 25(OH)D levels between 50–100 ng/mL is safe and cost-effective for disease prevention, ensuring optimal health outcomes.

## 1. Preamble

The Industrial Revolution increased indoor living and air pollution in urban settings. It reduced UVB exposure, especially in temperate climates in higher latitudes far from the equator, limiting vitamin D_3_ synthesis. Having darker skin color limits the vitamin D generations in the skin [1] and, thus, increases the vulnerability to infections [2,3]. During the past four decades, the dermatology community has warned the public about sun exposure, encouraging the overuse of sunscreens. This significantly contributed to the widespread hypovitaminosis D, which became a pandemic [4,5].

In parallel, in recent decades, a decline in mean serum 25-hydroxyvitamin D [25(OH)D] levels has been observed in many countries, reported in 19 European nations and the National Health and Nutrition Examination Surveys (NHANES) [6,7]. This decline parallels the rising incidence of metabolic disorders, such as obesity, type 2 diabetes, metabolic syndrome, infections, and sepsis. Additionally, all of these increase COVID-19-related complications, severity, and deaths [8,9].

The rising prevalence of severe vitamin D deficiency [defined as serum 25(OH)D <12 ng/mL] has led to an increase in the incidence of disorders like cancer [10], obesity and diabetes, infections, and sepsis [11,12,13,14], which cause over 11 million deaths annually [15]. Countries lacking effective supplementation guidelines or suboptimal/ineffective vitamin D recommendations reported lower population serum 25(OH)D levels. This increases the vulnerability and leads to higher incidences of chronic disease—obesity, diabetes, cancer, infection/sepsis rates, and escalating healthcare costs.

### 1.1. Establishing Optimal 25(OH)D Levels for Disease Prevention

Whereas, nations that prioritize public health and focus on vitamin D, like Norway, Sweden, Finland, and equatorial countries that are exposed to year-long sunlight, have higher population serum 25(OH)D levels, leading to fewer hospitalizations and lower all-cause mortality from SARS-CoV-2 [16,17,18]: thus lower healthcare costs. SARS-CoV-2 and other respiratory viral infection outbreaks are highest during winter when an individual’s vitamin D levels are lowest [19,20]. Studies have shown that COVID-19 vaccines have limited impact and failed to mitigate these outbreaks [19,21,22].

Maintaining the community’s vitamin D sufficiency reduces infections and sepsis and improves overall health. Data over the past four years revealed that introducing the COVID-19 vaccine did little to mitigate outbreaks or deaths from SARS-CoV-2 [22]. In contrast, maintaining vitamin D sufficiency reduces infections and sepsis, promotes overall health, and supports optimal bodily functions. The following terminology (definitions) were used in this SR to classify vitamin D status.

### 1.2. Definitions of Vitamin D Statuses

***(a)***  ***Vitamin D Sufficiency:***

A serum 25(OH)D concentration exceeding 50 ng/mL (with a physiological range of 40–100 ng/mL) is necessary to effectively address severe disorders like infection/sepsis, autoimmune diseases, and cancer. Considering the increased utilization of D_3_ and its metabolites in serious conditions like severe acute infections, higher serum 25(OH)D concentrations are necessary for effective management and better outcomes [23,24]. From a population and public health perspective, the minimum community-wide serum 25(OH)D concentration—vitamin D sufficiency—to mitigate most disorders is 40 ng/mL [25,26,27]. Despite recent outdated vitamin D guidelines that only focus on the skeletal effects of vitamin D [28], the emerging evidence confirms that the minimum level for individuals to cover the disorders mentioned is 50 ng/mL [25,29].

***(b)***  ***Hypovitaminosis D:***

Hypovitaminosis D is a broader term encompassing both vitamin D deficiency and insufficiency—a serum 25(OH)D concentration less than 40 ng/mL [26,30]. Below this, it is suboptimal to sustain its intended physiological and biological functions, especially in extra-skeletal and renal tissues [29]. Hypovitaminosis D exacerbates most health disorders and increases susceptibility to infections and autoimmune disorders [31], illustrating the need for higher circulating D_3_ and 25(OH)D concentrations [23].

***(c)***  ***Vitamin D Deficiency:***

Vitamin D deficiency is defined by serum 25(OH)D levels below 20 ng/mL [32]. This hypovitaminosis D elevates parathyroid hormone (PTH) levels, leading to secondary hyperparathyroidism, which contributes to additional disorders and increases susceptibilities, particularly to non-skeletal conditions. Similar impairments are observed when the circulating calcium-to-magnesium ratio (optimum around 2.0) exceeds 3.2 or falls below 1.5 [33]. Individuals with vitamin D deficiency respond well to appropriate supplementation, underscoring its therapeutic potential and the importance of including deficient subjects in clinical trials.

***(d)***  ***Severe Vitamin D Deficiency:***

Severe vitamin D deficiency is defined as a 25(OH)D concentration below 12 ng/mL, though some studies use a cut-off value of 10 ng/mL as the threshold. Most individuals with severe deficiency show signs and symptoms of neuromuscular and skeletal dysfunction [20]. In addition, vitamin D deficiency worsens several key diseases and increases their complications and mortality, especially from cardiovascular disorders, cancer, infections, septicemia, and premature deaths [23].

## 2. Systematic Review Procedure

This systematic review (SR) was designed to evaluate whether the reported relationships between vitamin D deficiency and the incidence and severity of SARS-CoV-2 infection and associated deaths contribute to and meet Bradford Hill’s criteria for causation.

### 2.1. Criteria Evaluated and Related Analyses of the Systematic Review

Another key point evaluated was the generalizability of clinical trial findings from trials. This depends on external validity and the ability to extrapolate to broader population groups, such as studies conducted in diverse settings, ethnic groups, and populations. The RCTs and meta-analyses (MAs) relevant to the topic reviewed in the SR showed considerable variation in study designs and quality. Some RCTs lacked explicit inclusion and exclusion criteria, while others had a biased selection without a proper rationale or hypothesis. Poorly designed studies, particularly those with questionable scientific intentions, the absence of a clear hypothesis, or flawed scientific concepts—such as those by Murai et al. and others—fail to provide reliable conclusions. Refs. [34,35,36,37,38,39,40]—compromised the reliability and robustness of the analyses, invalidating their conclusions.

### 2.2. Methods Used in This Systematic Review

We performed a comprehensive search, and the data was abstracted and synthesized. Peer-reviewed medical and scientific literature in English on the associations between vitamin D and infections, focusing on SARS-CoV-2, the virus responsible for COVID-19, was evaluated.

#### 2.2.1. Meta-Analyses: Addressing Selection Bias and Unscientific Study Designs

Considering these factors, the study ensured that the findings on vitamin D and its health benefits are robust, dependable, unbiased, and applicable to broader populations and settings. The following section discusses the methods used to compare the quality of evidence from poorly designed and conducted RCTs [41,42,43,44,45,46,47] with well-organized and prospective observational studies and RCTs [29,48].

#### 2.2.2. Data Sources and Search Strategy

The primary data source for this review was PubMed (www.pubmed.gov, accessed on 3 November 2024), supplemented by searches in other scientific databases, including Scopus, Web of Science, Medline, EMBASE, and the Cochrane Central Register of Controlled Trials. The search terms used included vitamin D, 25(OH)D, 1, 25(OH)_2_D, calciferol, calcifediol, calcitriol, clinical studies, RCTs, complications, clinical outcomes, COVID-19, and SARS-CoV-2, in different combinations. A thorough search was conducted across various research databases using keywords related to COVID-19 vaccines, mRNA vaccines, complications, immune evasion, vaccine efficacy, and alternative therapies like vitamin D and ivermectin.

Combining keywords narrowed the search to manageable results across databases like PubMed, Medline, Web of Science, and EMBASE from November 2019 to the end of 2024 (searches were performed at the beginning and end to capture all relevant studies). The focus was on clinical trials, RCTs, ecological/prospective studies, and relevant original and review articles, following systematic review methods [49,50]. References were selected, evaluated, and incorporated based on their relevance to the topic.

#### 2.2.3. PICOS Process

This SR followed and updated the PRISMA statement and guidelines and PICOS process (participants, intervention, comparison, outcome elements, and study design philosophies: PICOS)—these focused on the clinical questions described below. The author followed the guidelines from the Equator Network (www.equator-network.org/, accessed on 10 November 2024), the PRISMA statement [51], and the PRISMA-P checklist [52,53] (see Table 1 for the PICOS criteria)—eligibility criteria are included as a supplement to assess the quality of the literature in synthesizing data.

### 2.3. The Area Focussed in the Systematic Review

#### 2.3.1. Generalizability and Applicability of Data to Broader Population

This SR evaluated several factors to ensure the data’s generalizability to a broader population [36]. These factors include (A) consistency and strength of association across different ethnic groups and countries [37,38]; (B) geographical considerations—accounting for latitude’s influence on sunlight exposure–synthesis of vitamin D [39,40,41,42,43]; (C) population and ethnic diversity—verifying applicability across various demographic segments with diverse ethnic groups [37,44,45,46,47]; and (D) study Settings—distinguishing between community, outpatient, and hospital settings to assess vitamin D supplementation’s effects on various patient groups and healthcare environments [39,40,41,42,43].

The study addressed the following factors to ensure the applicability and generalizability of the findings to broader populations:

(A) Geographical Diversity—Incorporating studies from various countries and latitudes to account for differences in sunlight exposure and vitamin D synthesis.
(B) Ethnic and Population Diversity—Including diverse ethnic groups and populations to confirm relevance across demographic segments.
(C) Study Settings—Differentiating between community, outpatient, and in-hospital settings to evaluate the impact of vitamin D supplementation across diverse healthcare environments.
(D) Inverse Associations—Presenting evidence of an inverse relationship between serum 25(OH)D concentrations and disease vulnerability, severity, and mortality rates from infections, including SARS-CoV-2.

#### 2.3.2. Evaluation of Study Designs and Quality of Clinical Studies, Including RCTs

This SR also focussed on the clinical study–-design flaws prioritized to understand the reasons behind the failures of recent mega vitamin D-RCTs. They often reported equivocal results or no correlation between vitamin D levels and clinical disease outcomes. Significant flaws included insufficient doses, infrequent administration, short durations, or allowing control groups to take over-the-counter supplements, compromising the validity of these studies. Well-designed studies consistently demonstrated that adequate vitamin D supplementation significantly benefits the primary clinical outcomes in individuals with hypovitaminosis D across various diseases and ethnic groups, including infections and cardiovascular, metabolic, and inflammatory disorders.

#### 2.3.3. Mechanisms and Mechanical Insights

Because of the focus on SARS-CoV-2, the SR examined vitamin D’s impact on the immune system, highlighting the prevention of symptomatic disease, complications, and deaths. The SR also evaluated key mechanistic areas and identified: (A) Genomic and non-genomic effects, including membrane-mediated and autocrine–paracrine effects that enhance innate immune defenses and antimicrobial peptide secretion. (B) Mechanisms for reducing infection severity and deaths—sufficiency boosts innate immunity and modulates adaptive responses, and reducing infection severity minimizes tissue damage from overactive T-cells and autoimmune reactions. Regarding SARS-CoV-2 and other viral infections, the benefits of vitamin D derive from modifying inflammation and oxidative stress and controlling acute-phase reactions, which enhance recovery and reduce complications. These findings underscore the importance of the longer-term maintenance of sufficient 25(OH)D concentrations for robust immunity.

### 2.4. Results from the SR

The SR followed the methodology and protocol described in Section 1 to track the relevant publications and their data and conclusions. The selected keywords targeted RCTs and observational, ecological, and epidemiological studies from December 2019 to November 2024 [54]. Without a time limit, supporting studies were included. Study quality was assessed, and duplicates or less relevant papers were eliminated, forming the final catalog of 294 manuscripts [52].

#### Manuscript Selection, Screening, and Data Accumulation

The SR followed the specified methodology and protocol to identify publications related to the topic. Keywords used to search for manuscripts on RCTs, observational, ecological, and epidemiological studies from December 2019 to November 2024 (COVID-19 period) [54]. Supporting laboratory and animal studies were included to strengthen the justifications. Study quality was assessed, and duplicate or less relevant papers were excluded, resulting in the final catalog [52].

**Figure 1:**
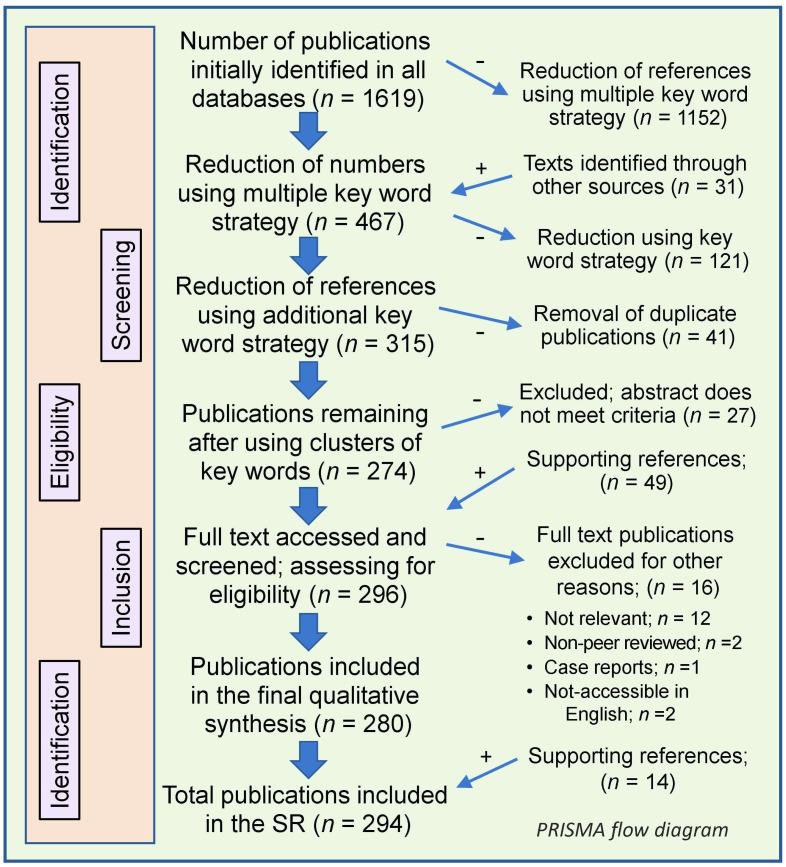
PRISMA flow chart—the process of selecting references aimed to address the importance of better-designed RCTs for advancing the knowledge of vitamin D, focusing on clinical study design errors and their elimination.

The initial screening identified 1619 manuscripts from combined databases. After excluding 1,331 unrelated or duplicate papers, 294 eligible articles were selected for this systematic review using the EndNote 21.1 reference manager. The study adhered to the process outlined in the Preferred Reporting Items for Systematic Reviews and Meta-Analyses (PRISMA) statement and guidelines [52,53,54] and the PICOS process: the summary data are included in Figure 1.

### 2.5. Scope and Synthesis of Systematic Reviews on Vitamin D and COVID-19: Data and Limitations

The SR followed the evidence-based PICO process—patient problems, interventions, comparison/control, and outcomes. A rationale with eligibility and exclusion criteria was developed [24]. The study examined potential biases in individual studies, including RCTs and meta-analyses, while noting their study design flaws [52,53]. The strength of evidence on the biology and physiology of vitamin D was assessed and categorized into immunity, autoimmunity, and infection control. Non-peer-reviewed and non-English publications were excluded.

A narrative conclusion is presented based on the analysis of the results and clinical outcomes [52]. The study protocol followed a systematic approach to identify, evaluate, and synthesize the study protocol to identify and evaluate. The study protocol followed a systematic approach to identifying, evaluating, and synthesizing the literature on the relationship between vitamin D and SARS-CoV-2. It explored whether hypovitaminosis D is a causative factor that increases the risk of infection, complications, and mortality. Unlike other reviews, this search focused on the COVID-19 pandemic from November 2019 onward, with a four-year, focused review.

### 2.6. Study Findings

This SR confirms a strong association between vitamin D deficiency and increased susceptibility to complications and deaths from SARS-CoV-2. Of the 329 clinical trials that used vitamin D/calcifediol in SARS-CoV-2 (https://c19early.org/dmeta.html, accessed 10 November 2024), only 11 showed no benefit—all the negative studies had significant design flaws [55]. The study also identifies poor-quality RCTs and meta-analyses that included such RCTs, which hinder proper conclusions about vitamin D deficiency and SARS-CoV-2 outcomes [21]. The SR also highlights the failure to approve early generic therapies like vitamin D and ivermectin [56], which could have reduced hospitalizations and deaths, reflecting poor policies and flawed advice from health organizations [21,56].

Since vitamin D is a threshold nutrient, its efficacy is best evaluated through large-scale, well-designed, longer-term observational or ecological studies rather than RCTs. The latter is unsuitable due to their pharmaceutical-based study designs and the impossibility of recruiting truly vitamin D-deficient participants [24]— those unexposed to sunlight or taking supplements [57]. Poorly designed RCTs and their inclusion in meta-analyses amplified faulty conclusions [32], fueling misinformation and inappropriately spreading skepticism about vitamin D’s role in health, particularly during the pandemic [19,22].

An evidence-based PICO process was applied to the SR, establishing the eligibility and exclusion criteria, biases in RCTs and meta-analyses, and flawed study designs [52,53]. In addition, the evidence on vitamin D’s biology and physiology was assessed, focusing on immunity, autoimmunity, and the ability to control infection, excluding non-peer-reviewed publications that were not in English. The findings highlight that maintaining serum 25(OH)D levels above 40 ng/mL reduced infection risks and severity, particularly for COVID-19. All the well-designed and conducted studies confirm the benefits of vitamin D. This SR underscores the critical role of vitamin D sufficiency in disease prevention. Over 300 clinical trials [55] strongly support an inverse association between serum 25(OH)D levels and COVID-19 severity, hospitalizations, and mortality [31,58,59,60,61,62,63,64,65,66,67,68,69,70,71,72,73,74,75,76].

**Figure 2:**
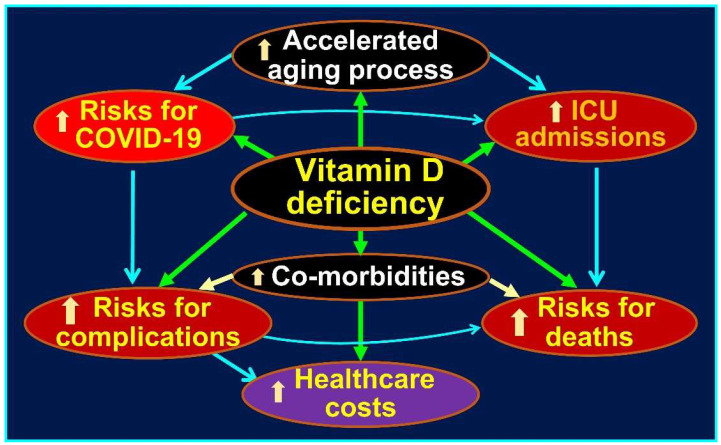
Vitamin D deficiency increases the aging process, debility, and co-morbidities, as well as the incidence and severity of infections. The red circles indicate the vulnerability, compilations, and deaths from SARS-CoV-2.

Clinical outcomes consistently correlate with study quality and design flaws. Well-designed and statistically powered RCTs have consistently shown positive clinical outcomes [77,78], whereas studies with inferior designs have consistently yielded negative or non-conclusive outcomes [42,79,80,81,82]. Advanced age, strongly associated with hypovitaminosis D, was a critical factor in increasing vulnerability to SARS-CoV-2. Based on the understanding from this research, Figure 2 illustrates multi-dimensional influences like aging, accelerated by co-existing vitamin D deficiency (and low ACE-2 levels), and the heightened adverse effects of SARS-CoV-2 [83].

## 3. Introduction

Over the past three decades, more than 50,000 research articles on vitamin D have been published in PubMed and other research databases. Those articles investigated vitamin D status and disease risks and confirmed a robust inverse association between serum 25(OH)D concentrations and disease vulnerability, severity, and death rates from various diseases. Out of these, over 1000 articles focussed on infections [8,9,31,84,85].

Despite the vast published literature, health agencies and some clinical societies/government-appointed committees continue to neglect the importance of natural defense mechanisms [56] and natural immunity [86] and the cost-effectiveness of repurposed, widely available economic agents available without prescriptions [19,21,56]. The latter group demonstrated longer-lasting, more robust immunity without adverse effects [19,83] than COVID-19 vaccines [87,88]. This situation worsened as health agencies focused primarily on COVID-19 vaccines and relied on them to overcome the pandemic, which led to increased COVID-19 deaths [73,89]. This failure prompted the present SR to investigate the critical factors that increase vulnerability to this coronavirus.

### 3.1. Benefits of Maintaining Steady Levels of Vitamin D and 25(OH)D for Infections

The beneficial role of maintaining vitamin D sufficiency is crucial for its biological and physiological functions. However, larger disease-specific datasets were published only recently, shedding light on the specific minimum serum 25(OH)D concentrations necessary to combat infections [29,90,91,92]. As with blood pressure, lipids, and hormonal levels, measuring circulating 25(OH)D (the only reliable method to assess vitamin D status) [93] is essential in clinical practice and RCTs.

There is no scientific evidence to confirm that minimum adequate vitamin D levels vary across different ages, population groups, or conditions. However, evidence confirmed that the minimum levels of serum 25(OH)D concentrations significantly vary based on tissues and disease [29,31,32]. In addition, micronutrient-related physiological and biological processes are not different in ethnic or age groups [94,95]. Based on the reported clinical trials, it is unsurprising that a 30 ng/mL level only benefits approximately 40% of disorders [32,96]. The minimum level (lower threshold), 50 ng/mL, applies to all individuals and disorders—all ethnic groups and conditions [32]. It will prevent over 99% of human disorders [20,23].

The mentioned assessment is recommended, particularly for those who are vulnerable, immune compromised, or have comorbidities [32,97]. In this regard, the 2024 Endocrine Society guidelines represent a significant error, among other errors [24,27], by recommending against the measurement of 25(OH)D [28]. Several leading vitamin D research groups refuted this erroneous guidance [27,98]. Demay et al.’s recommendation [28] even failed to advocate the target 25(OH)D level of 30 ng/ mL (75 nmol/L) recommended in the 2011 guideline [97], so it can only harm people.

The Endocrine Society guidelines (2024) focused on vitamin D requirements in healthy people’s skeletal systems, not on other systems, vulnerable groups, disease statuses, or sick patients [28]. Therefore, such recommendations are impractical and should not be generalized or used for designing clinical studies related to extra-skeletal tissues, policy-making, or clinical practice [27,98]. Additionally, the 2024 report ignored the vast amount of data on the extra-skeletal benefits of vitamin D published over the past 15 years [27,32]. Others have emphasized the importance of rehabilitating those with complications after post-COVID syndrome [99,100].

Some claim that “in observational studies, exposure to a nutrient like vitamin D does not cause alter risks.” This false argument contradicts key public health principles—“an intervention reduces the risk of disease or disorder”—confirming a causal link. Along with its genomic effects, various other mechanisms of vitamin D-mediated signaling have been described, including direct actions on membrane stabilizations [101,102] and the autocrine and paracrine signaling pathways stimulating the immune system [103,104,105]. These mechanisms show that vitamin D regulates immune activity through intracellular signaling —autocrine and paracrine effects [25,103,104] and genomic functions [106,107], helping prevent viral replication, promote viral destruction, and protect against tissue damage caused by overactive T-cells and autoimmune reactions [108,109,110].

### 3.2. Benefits of Adequate Vitamin D Supplementation in Infections

The vital role of vitamin D adequacy in combating acute infections was confirmed a decade ago [91,111,112]. Nevertheless, it was only recently that the serum 25(OH)D concentration thresholds needed to overcome infections [58,113,114] and reduce other health risks [25,59,60,115,116,117,118] were established, including in children [61,114,119,120]. Meanwhile, the clinical benefits following direct sun exposure from ultraviolet B (UVB) rays in people with tuberculosis and psoriasis were recognized over 100 years ago, with increased survival [121,122]. The severity of tuberculosis, its spread, and deaths were significantly higher in those with hypovitaminosis D [73,123].

Subsequent studies affirmed these findings and explained the mechanisms by which UVB and vitamin D supplements aid in faster recovery [124,125]. Data from numerous studies consolidated these findings [17,90,91,126]. The converging data strongly supported that the minimum effective serum 25(OH)D concentration to reduce infections and their severity is 40 ng/mL (125 nmol/L) [31,60,63], while optimal levels are above 50 ng/mL [17,29,48,91,112,127,128]. Numerous studies have reported that properly using vitamin D_3_ and calcifediol significantly reduced complications and deaths from SARS-CoV-2 infection in those with hypovitaminosis D [62,64,65,73,129,130,131,132].

A large meta-analysis encompassing a variety of heterogeneous studies concluded that even with relatively low doses of vitamin D, there is a reduction in the incidence of acute respiratory illnesses [58,133]. The same researchers subsequently published articles, including RCTs, asserting that vitamin D significantly reduces acute upper respiratory tract infections [17,35,134,135]. They and others reported that intermittent dosing regimens were ineffective [14,37]. In contrast, a recent meta-analysis reported no difference between daily vs. intermittent vitamin D administration, up to once a month [136].

### 3.3. Evidence Related to Respiratory Viral Infections, Including SARS-CoV-2

The published studies reported strong inverse associations between vitamin D intake, serum 25(OH)D concentration, and reduced viral respiratory infection rates [58,133,134,137] and both the severity of and mortality from COVID-19 [62,67,69,138,139]. However, these studies and the use of vitamin D/calcitriol for SARS-CoV-2 infection have also been neglected [60,65,69,70,71,72,73,74,129,132,140,141,142,143,144,145,146,147]. Over 121 peer-reviewed clinical studies that used vitamin D as the primary intervention to investigate the effects on clinical outcomes in COVID-19 were published between January 2020 and October 2024 (https://c19early.org/d and https://c19early.org; accessed 10 November 2024) [55]. This website objectively compiles all negative and positive vitamin D and COVID-19 studies up to May 2023 [55].

The website https://c19early.org (accessed on 10 November 2024) provides an unbiased, independent, and comprehensive dataset with real-time meta-analyses available for free [55]. https://c19early.org (accessed on 10 November 2024) reports all the other generics and patented agents tested for prevention and adjunct therapies against SARS-CoV-2. Summarizing data from 4,429 COVID-19-related clinical studies with real-time meta-analyses and forest plots [55], it is the largest comprehensive, freely available, and reliable resource worldwide. The site includes observational and prospective studies, RCTs, systematic reviews, and MAs. Given the suboptimal quality and bias in many recent meta-analyses [55], this resource ensures reliable data synthesis for understanding vitamin D’s impact on COVID-19 and other health outcomes.

The evaluation criteria for this SR included internal validity (reasonable study design and proper execution), consistency (evidence volume, trend, and outcomes), and magnitude of the effect (larger effect sizes with narrower 95% confidence limits) [24]. Clinical trial (especially RCTs) data should be presented as “absolute” values rather than the less reliable “relative” effects often used by pharmaceutical companies to exaggerate the efficacy of drugs, such as antiviral agents and mRNA-based COVID-19 vaccines [19,22]. External validity reflects on whether effects were observed across different settings, generalizability to diverse populations, whether known mechanistic effects of activated vitamin D were relevant to the health issue studied, and whether the data and conclusions are practical and make good judgment—i.e., scientific and common sense approaches [148].

Ecological and prospective observational and retrospective studies, randomized controlled clinical trials, systematic reviews, and meta-analyses relevant to vitamin D and SARS-CoV-2/COVID-19 were examined. This SR provides a robust evidence base supporting the beneficial effects of vitamin D supplementation in reducing infection severity, particularly in the context of COVID-19. Clinical trials reported an inverse correlation between serum 25(OH)D with severity and mortality from SARS-CoV-2 [149]. Others reported that a moderate dose of vitamin D of 60,000 IU/week relieves post-COVID syndrome symptoms, including fatigue and anxiety, and improves cognitive symptoms [27,150].

## 4. The Importance of Proper Designs of RCTs

While many studies have reported beneficial clinical outcomes in inflammatory disorders [95,151,152,153] and infections [35,134,154], not all the studies have confirmed these beneficial effects on primary clinical outcomes [34,42,133,155,156]; this is predominantly due to poor study designs [24]. Another critical issue is the failure to recognize vitamin D as a threshold nutrient [23,32]. Consequently, RCT-principle-based clinical trial protocols designed to obtain pharmaceutical agent approvals are unsuitable for testing micronutrients/nutraceuticals like vitamin D [23]. Traditional RCT designs are fundamentally limited by seizing and the complexities of nutrients/nutraceuticals [157]. It is also important to note that the benefits of vitamin D, similar to other micronutrients, rely on maintaining steady blood levels above a specific threshold with minimal fluctuations.

### 4.1. Conflicts of Interest and Study Design Errors

Despite the vast literature, primary outcomes from some larger (mega) clinical studies [41,42,43,44,45,46,47] published over the past 15 years reported either equivocal or no correlations [10,42,158]. This trend worsened during the past decade, with some studies displaying obvious and well-understood study design errors—some RCTs appeared “designed to fail” [82,95,159,160,161,162,163]. The study design errors of these mega RCTs [27,41,42,43,44,45,46,47] include recruiting vitamin D-replete subjects, compromising their validity and conclusions [82,95,159,160,161,162,163].

Ironically, big pharma designed and funded many of these negative studies, even though they were conducted at academic institutions [41,42,164]. The mainstream media gave disproportionate publicity to these negative studies, adding to the confusion on vitamin D research. This negative publicity created uncertainty about the value of adequate vitamin D in reducing health risks for COVID-19 and other disorders. These are discussed in detail below. The following section addresses Bradford Hill’s evaluation criteria regarding vitamin D status and SARS-CoV-2 infection.

### 4.2. Hill’s Criteria—Linking Hypovitaminosis D to COVID-19 Clinical Outcomes

Cohort studies propose a reverse causation hypothesis, suggesting COVID-19 causes hypovitaminosis D, though unproven. Samaha et al. observed that symptomatic disease increases vitamin D consumption [66]. However, most studies confirm that vitamin D-deficient individuals are more susceptible to symptomatic disease and complications due to weakened immunity [25,165]. Samaha et al. also noted that symptomatic infection rapidly depletes vitamin D levels. Without supplementation, serum concentrations drop to deficient levels, potentially prolonging recovery and increasing complication risks [22,66].

For those who are symptomatic, in the absence of supplementation with vitamin D, patients are likely to become sicker and succumb to SARS-CoV-2 or have a prolonged recuperation [19,66]. This situation is avoidable by providing sufficient daily vitamin D supplements [23,166] or single or multiple doses of calcifediol [29,68,75,141,167,168] (see below for details). Robust evidence presented in the SR concludes that serum vitamin D status is a biological determinant associated with SARS-CoV-2 infection and related outcomes. Table 2 illustrates the factors in Hill’s criteria that should be satisfied to conclude causation.

### 4.3. Importance of Real-Time Meta-Analysis to Understand the Efficacy

Since vitamin D is a threshold nutrient, data revealed that circulating 25(OH)D needs to be above the minimum adequate level of 40 ng/mL to have a robust immune system [23,195,196,197] to prevent symptomatic disease and complications [62,74,165]. Higher serum 25(OH)D concentrations (e.g., between 50 and 100 ng/mL) are associated with significantly less symptomatic infection [23,74,198] and fewer hospitalizations and deaths from SARS-CoV-2 [23,27,73,98,198].

A real-time meta-analysis of 321 vitamin D and SARS-CoV-2-related clinical studies (122 treatment and 199 observational studies) revealed a statistically significant reduction in hospitalization, ICU admissions, and mortality rates (https://c19early.org/dmeta.html#perspective, accessed on 25 November 2024) [55]. Therefore, the repetitive call by authors for “more RCTs and meta-analyses” lacks merit. Considering the different half-lives, administering a combination of calcifediol for immediate effects and D_3_ for medium-term benefits offers optimal clinical outcomes [25,29]. This approach enhances protection against infections like sepsis and acute viral infections. It significantly reduces complications and deaths associated with COVID-19, as corroborated by the positive findings of 318 (out of 329) vitamin D clinical studies [55].

## 5. Clinical Trial Design Failures Led to Erroneous Data and Conclusions

This study data revealed that many clinical trials, especially RCTs, investigated the relationship between vitamin D status, infections, and other conditions. However, some did not have a hypothesis, and others failed to test whether hypovitaminosis D is a causative factor [159,160,161,162]. The failed studies did not use hard endpoints, like hospitalization, ICU admission, or mortality, as the primary outcomes and depended on symptomatology. Other trials administered insufficient vitamin D or a single bolus dose [32] without follow-up daily supplements to severely ill patients, particularly those in the ICU, treated late in the disease course [34]: typical study design errors. Unsurprisingly, such trials uniformly failed.

Most studies failed to measure baseline serum 25(OH)D levels or ensure the participants were deficient. Others failed to measure the 25(OH)D levels achieved in circulation (i.e., the desired therapeutic concentrations to overcome the disorder). In addition, SARS-CoV-2 infection increases the consumption of calcitriol within immune cells and increases the intake/diffusion of D_3_ and 25(OH)D from the circulation. Without D_3_ supplements, serum 25(OH)D levels decrease from symptomatic infection [66,199]. Combining these reduces the immune capacity and delays the recovery and causality determinations. Calcifediol instead of vitamin D can eliminate some of these errors and decrease clinical responses [27].

### 5.1. Objectives of Clinical Trials Should Be:

Clinical trials are desinged to test hypotheses, assess a drug’s efficacy, or evaluate a medical device with the view of obtaining regulatory approvals. Safety and dose responses are examined in Phases 1 and 2, while Phase 3 trials assessed the efficacy. Phase 4 studies expand a drug’s use post-approval and seek regulatory approval for other conditions. Proper randomization into active, control, or placebo groups achieves the trial’s objective of having comparable groups to test. The methods to achieve that goal include random number generation, one-to-one, block randomization, stratification, and intent-to-treat principles. Study groups must remain balanced apart from active interventions.

Randomized studies can be single-blinded or double-blinded, prospective, and may include an active comparator (one or more groups with different doses) and a control group. While well-conducted RCTs offer higher validity and reliability, they are more complex and expensive than case-control or cohort studies [200]. Nutrient studies present additional challenges compared to pharmaceutical RCTs [23].

Pharmaceutical-based RCT designs or intention-to-treat approaches are often unsuitable for analyzing nutrient efficacy in most trials [82,201]. As discussed, the RCT methodology, in general, is unsuitable to test nutrient-related hypotheses. Efficacy, safety, doses, practicality, common sense, and benefits of nutrient usage should be examined using community-based well-designed observational studies.

### 5.2. Lessons Learned from Large Pre-Pandemic Vitamin D RCTs

As discussed above, recently published large RCTs conducted before the SARS-CoV-2 pandemic exhibited many design flaws. In summary, in RCTs with failed primary endpoints, such as the VITAL study, most subjects at the enrolment were not vitamin D deficient, allowing them to continue taking over-the-counter nutrient supplements [42,47,159,160,161,162,202], especially the control group. Any of the errors mentioned above would invalidate the conclusions of such an RCT [82,95,159,160,161,162,163].

The studies that used vitamin D intake as treatment but failed to measure baseline status or changes in serum 25(OH)D levels lacked sufficient statistical power to detect differences between intervention and placebo groups [41,42,43,44,45,46,47,163]. Another confounder is magnesium deficiency [33], which is essential for hormone synthesis, calcitriol VDR interactions, and reducing complications and mortality in post-COVID syndrome [203,204].

The failed large vitamin D-related RCTs mentioned above unrelated to SARS-CoV-2 [42,43,44,45,46,47] had design shortcomings and conflicts in study designs [205], and their implementation obscured the relationships between vitamin D status and disease states, further contributing to the failures and confusion [25,29,82,164,206]. Many investigators mistakenly relied on the administered vitamin D dose for analyses and correlations [82,207]. In contrast, well-designed and properly conducted clinical studies for an appropriate duration across various countries have consistently supported the beneficial primary endpoints of vitamin D supplementation in individuals with hypovitaminosis D, like reducing COVID-19 illness rates [35,95,126,134,151,152,153,154,208,209,210,211,212,213].

### 5.3. Key Causes of Failures in Vitamin D RCTs

Despite the large bolus doses administered, these studies showed that the median serum 25(OH)D achieved with vitamin D was less than 30 ng/mL, which is grossly insufficient to overcome an infection. The negative outcome illustrates that patients who are severely ill would not benefit from early therapies [34,35,36,37,39,40,214,215,216,217]. Data showed that the minimum should be 50 ng/mL to overcome viral infections [29,90,112,127]. Nevertheless, the key is to administer sufficient doses of vitamin D as early as possible. This fundamental principle was illustrated by comparing 50,000 IU vs. 400,000 IU in high-risk older patients—a higher bolus dose had significantly reduced complications and mortality from SARS-CoV-2 by day 14 [38].

When daily doses do not follow bolus doses and fail to maintain therapeutic serum 25(OH)D levels, higher serum 25(OH)D concentrations on admission are associated with lower pulmonary involvement, shorter hospitalization, and fewer ICU admissions [37]. In all 11 negative studies, the authors overlooked the biological physiology of vitamin D and its role in immune system maintenance. These design failures led to adverse outcomes and claims that vitamin D had no benefit.

Administering a large dose of vitamin D to acutely ill patients is unlikely to sustain therapeutic serum 25(OH)D levels beyond two to three weeks due to poor gastrointestinal absorption and rapid utilization during infection-related immune processing. Therefore, bolus doses may not maintain D_3_ and 25(OH)D concentrations or achieve the intended outcomes, like reducing ICU admission or mortality beyond two weeks [38]. However, clinical trials have confirmed no adverse effects from administering one-time doses of 60,000 IU daily for seven days [218], 300,000 IU [214], or 600,000 IU as bolus doses in adults and children [34,216,219]. Such loading doses should not be repeated and must followed with a suitable daily dose of vitamin D.

### 5.4. The Ways to Minimize Study Design Errors

Section 4.1 and Section 4.2 summarize the fundamental steps for designing a nutrient clinical trial. Many of the errors mentioned are easily avoidable but are repeated in large-scale vitamin D trials [42,43,44,45,46,47]. Avoiding these design errors is key to achieving meaningful outcomes [27]. Understanding the biology and physiology of vitamin D and its immune modulation mechanisms is essential before designing a trial. Based on the published evidence, institutional review/ethics boards (IRBs) should not have approved the failed clinical trials (3% of all the trials), as their unethical designs could have harmed participants [42,43,44,45,46,47].

A neglected area in nutrient clinical trials is correcting cofactor deficiencies, such as magnesium, zinc, selenium, boron, and other trace minerals, which can confound results. For example, magnesium sufficiency, even with vitamin D, is linked to reduced complications and mortality in post-COVID syndrome [220], highlighting the importance of cofactor sufficiency for better outcomes. Adequate levels of these cofactors are vital for enzymatic reactions, hormone synthesis, release, and calcitriol’s interaction with vitamin D/calcitriol receptors (VDRs/CTRs) [203,204].

As discussed, well-designed, statistically powered RCTs that recruit vitamin D-deficient participants and investigate vitamin D as a primary intervention for SARS-CoV-2 infection or infection would invariably generate positive clinical outcomes. Error-free study designs would ensure robust primary endpoints, such as hospital length of hospital stay, ICU admissions, and mortality [24]. The target serum 25(OH)D level may vary by disease and should be maintained daily rather than through bolus supplementation, except at study entry [35,37,221,222,223].

For infections (like SARS-CoV-2 and tuberculosis) [25,29,48,91,112], cancer, autoimmune diseases, and longevity [25,29], the goal should be to achieve and sustain serum 25(OH)D levels above 50 ng/mL, with a range of up to 80 ng/mL. The actions of calcifediol are swifter than those of vitamin D [224,225,226]. All the clinical trials using calcifediol in SARS-CoV-2 have reported statistically significant improvements with hard endpoints [68,70,75,141,167,168]. In contrast, poorly designed studies, as mentioned earlier [41,42,43,44,45,46,47], characterized by flawed criteria illustrated in Section 4.1 and Section 4.2, are unlikely to demonstrate favorable clinical outcomes [221,222].

### 5.5. Faulty Study Designs Mislead Vitamin D–SARS-CoV-2 Trial Conclusions

As discussed, nearly all the vitamin D-related clinical trials reporting no association between vitamin D status and infections—or conditions such as diabetes, obesity, and cancer [41,42,43,44,45,46,47]—were poorly designed to test whether hypovitaminosis D is a causative factor [159,160,161,162]. Many failed to recruit vitamin D-deficient subjects or use meaningful solid endpoints like hospitalization, ICU admission, or mortality as the primary outcomes [214], initiated treatment too late in the disease, or failed to measure serum 25(OH)D levels [32]. Additionally, many studies assessed the treatment effect based on vitamin D intake, ignoring variations in response due to the baseline status and body weight. Instead, achieved serum 25(OH)D levels should be measured [82,161,227].

A good RCT should recruit participants with vitamin D deficiency to use the nutrient as the primary intervention in SARS-CoV-2 infection, with the interventions started at the earliest possible time (i.e., on admission) [19,22,228,229,230]. Such trials must have sufficient statistical power (demonstrated by Power Analysis) and use hard primary endpoints. The target level may vary based on the disease investigated [23,166] and is used daily rather than in bolus supplementation [35,37,221,222,223]. Therefore, poorly designed studies with the mentioned flawed criteria are unlikely to generate favorable clinical outcomes [221,222]. In parallel, such studies must be excluded from systematic reviews and meta-analyses to avoid misleading data and erroneous conclusions [23].

### 5.6. Failed COVID-19 Pandemic—Related Vitamin D RCTs

By late 2020, it was well-established that a single large bolus dose of vitamin D, without daily or weekly maintenance doses [17] or infrequent repeated high doses, does not benefit individuals with hypovitaminosis D who are seriously ill, including those with SARS-CoV-2 infection (see Section 4) [34,35,36,37,38,39,40]. It was understood that early therapies, such as vitamin D and ivermectin, are most effective as prophylactic and in the early stages of infectious diseases [228,229,230]. These agents are ineffective when administered in the late stages of the disease [20], primarily because it takes more than a week for absorption and conversion into 25(OH)D, especially in sick individuals [25,214].

Additionally, the studies confirmed that vitamin D deficiency impairs the ability to combat infections, mainly intracellular bacterial and viral infections [16,17,18,231]. Table 3 discusses all the failed vitamin D–SARS-CoV-2 trials that consistently had significant study design flaws [24]. In addition to the failed clinical trials, Table 3 illustrates three positive studies with similar protocols but administered earlier in the disease.

The protocols in Table 3 illustrated that administering vitamin D, despite higher doses, to severely ill, late-stage patients with SARS-CoV-2 did not improve the condition or deaths [21,56]—a phenomenon also exhibited with ivermectin in COVID-19 [233]. The pharmacokinetics of high bolus doses of vitamin D, without follow-up higher daily doses, suggest that ongoing treatment spread over time is more effective [234]. At least in theory, there is a possibility, especially with repeated bolus doses, that they could induce 24-hydroxylase enzymes (from the CYP24A1 gene) and fibroblast growth factor-23 (FGF23) (which may remain elevated for several weeks), which reduces the levels of vitamin D and its metabolites [23,198].

## 6. Enhancing Natural Immunity to Overcome SARS-CoV-2 Infections

A “real-time meta-analysis” of 329 clinical studies on vitamin D and COVID-19 (comprising 124 treatment/interventional and 205 observational studies) (except for the 11 flawed clinical trials) reported statistically and clinically significant improvements in complications, hospitalization, ICU admissions, and mortality [19,22,235]. Based on potencies and half-lives, the combination of calcifediol (for immediate effect) and D_3_ (for medium-term effects) will maximize protection against infections [23,31,166] and sepsis [236] and significantly reduce COVID-19-associated complications and deaths [55,73,237].

Despite claims, infections can be overcome by stimulating (boosting) the immune system [25,234]. Since the vigor of the immune system primarily depends on vitamin D (and other micronutrients and cofactors), its supplementation is a critical intervention. Considering this, emergency rooms, hospitals, and medical protocols should include administering vitamin D on the first encounter—whether exposed, infected, symptomatic, or those who have developed complications [23,166].

### 6.1. Validated Disorders Associated with Vitamin D Deficiency Based on Hill’s Criteria

Ample evidence has been reported demonstrating that low vitamin D status increases the vulnerability and the rates of infections, complications, and mortality. Examples include hospital-acquired infections [91,112], tuberculosis [238,239,240], viral respiratory illnesses in children and adults [58,133,134], and SARS-CoV-2 [69,70,141,171,241,242,243]. Studies related to SARS-CoV-2 demonstrated that pre-existing vitamin D deficiency increases the risks of SARS-CoV-2 infection [60,74,147] and complications [71,73,147], hospitalizations [65,72,144,145,146], and deaths [71,129,132,142,143]. Administering the correct doses and frequency of vitamin D supplements in deficient persons significantly reduces infection risks, complications [237,244], and deaths from SARS-CoV-2 [60,65,71,72,73,74,129,142,143,144,145,146,147].

For a nutrient RCT to substantiate Bradford Hill’s criteria, a supplement intervention must be provided to people with proven vitamin D deficiency (i.e., by measuring serum 25(OH)D; mandatory entry criteria—(see below for details and exceptions), with a matching placebo to the control group [190]. Those studies adhered to Heaney’s criteria by recruiting vitamin D-deficient subjects and validated Hill’s criteria for several disorders, including cancer [161,245,246,247,248], periodontal disease [176], cardiovascular risk factors/disorders [249], and multiple sclerosis [250,251].

In addition to its genomic effects via the binding of calcitriol with its receptors, a crucial mechanism involved in non-musculoskeletal systems [252] is the sufficient generation of intracellular calcitriol with target cells, like immune cells, vital for intracellular vitamin D signaling [104,193,253,254,255,256]. It supports further biological plausibility [64,91,142,161,188,257,258]. This autocrine/intracrine and paracrine signaling provides additional beneficial effects of vitamin D, reducing complications [244,259,260] and deaths from SARS-CoV-2 [31,58,59,60,61,62,63,64,65,66,67,68,69,70,71,72,73,74,75,76,129,142,143,144,145,146,147,188,189].

A significant oversight in vitamin D studies is the failure to provide essential cofactors for optimal vitamin D and calcitriol functions [23], primarily magnesium [261,262]. Other critical cofactors include antioxidants, trace minerals (boron, zinc, and selenium), vitamins A, B_2_, C, and K, resveratrol, and omega-3 fatty acids [263], depleted during immune responses, infections, and autoimmune disorders. Supplying these cofactors, especially magnesium, to trial participants, including controls, is crucial [33]. Most vitamins and cofactors can be provided cost-effectively via a balanced diet, multivitamins, and trace-element supplements [263,264]. Ethical concerns can be mitigated by giving placebo groups 600 IU of vitamin D while restricting unnecessary supplements. Table 3 links hypovitaminosis D to various diseases per Hill’s criteria [148].

### 6.2. Vitamin D Deficiency and SARS-CoV-2 Risk: Evidence Supporting Bradford Hill’s Causation Criteria

**Figure 3:**
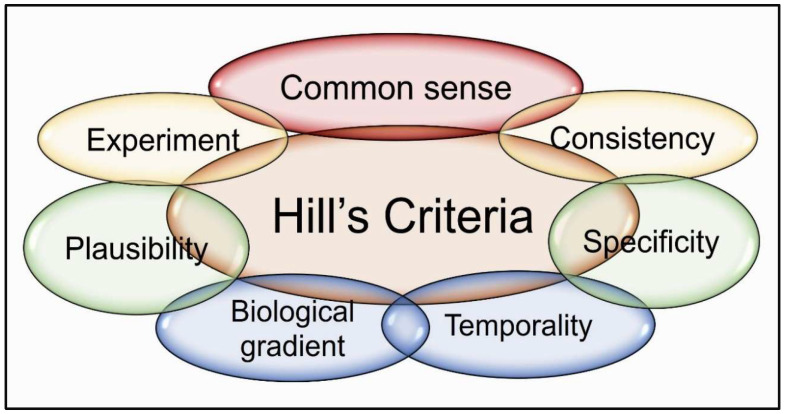
Illustration of seven criteria to be satisfied to establish causation. However, not all these criteria need to be met. In addition, they do not work smoothly without applying common sense.

Recent data from well-conducted, adequately powered trials in vitamin D-deficient subjects support the efficacy of vitamin D in SARS-CoV-2, showing positive outcomes when proper doses (preferably daily) were used [201]. Over 120 such clinical trials using vitamin D in deficient participants early in the disease, ensuring dosing regimens maintained target serum 25(OH)D levels (above 50 ng/mL) provided significant benefits [25,48,128]. Proper study designs are essential to confirm associations and causation, achieving health benefits after correcting deficiency [163,265,266]. Hill’s criteria for causality are summarized in Figure 3.

### 6.3. Do We Always Need RCTs to Establish Efficacy and Causality?

Using RCTs, meta-analyses, and observational studies, researchers have applied Bradford Hill criteria to demonstrate how hypovitaminosis D increases various disease risks. These studies employ a variety of methodologies, such as RCTs, meta-analyses, and observational cohort studies, to explore the link between vitamin D deficiency and different diseases. However, history provides numerous examples where observational data alone confirmed the causality, such as when RCTs were impractical, unethical, or unnecessary for identifying the root causes or preventing diseases.

Unlike pharmaceutical interventions, in many cases related to nutritional deficiencies or environmental factors, conducting an RCT is impractical or unethical, making alternative study designs more suitable for establishing causality and guiding public health measures [24]. Table 4 illustrates examples of common-sense observational data and public health approaches that have effectively identified and eliminated public health risks without the need for RCTs.

### 6.4. Why Are RCTs Unsuitable for Testing Micronutrient Efficacy?

Most public health policies, particularly micronutrients and nutraceuticals, are based on observations rather than RCTs (Table 3). Groundbreaking discoveries, such as nutrient deficiencies, acute diseases, and behavioral disorders, often stem from observational studies rather than RCTs or meta-analyses. RCTs, meta-analyses, and Mendelian randomization should not be the primary methods for testing micronutrient efficacy.

RCTs were designed initially to evaluate synthetic drugs (pharmaceuticals) for regulatory approval. However, applying these principles to micronutrients is unsuitable for assessing their efficacy. Key differences between pharmaceuticals and micronutrients, such as pharmacokinetics, dose-response behavior, and difficulty creating nutrient-free control groups, complicate micronutrient research. In industrialized nations, most RCT participants already consume over-the-counter supplements like vitamin D, with mean serum 25(OH)D concentrations around 30 ng/mL [42,283]. Factors like variable sun exposure and unquantified dietary intake make establishing a proper vitamin D-deficient control group impossible.

Instead of RCTs, testing threshold nutrients like vitamin D should be conducted through longer-term observational or ecological studies. Properly designed observational studies have consistently shown that low 25(OH)D levels increase susceptibility to various disorders and their severity, thereby establishing both efficacy and causality in addressing hypovitaminosis D. Compared to observational studies, RCTs are more expensive, complex, and time-consuming [205,284]. Along with recent, conflicted, poorly designed vitamin D-related mega RCTs that led to negative outcomes [42,285,286,287,288], taxpayer funds should not be used for future vitamin D mega trials.

RCTs are not always necessary or practical for identifying causative factors in environmental or health issues (Table 3). Examples include hypovitaminosis D in acute life-threatening conditions and behavioral disorders. Observational, prospective, and retrospective studies are cost-effective, easier to design, and often yield more accurate outcomes close to causality. Identifying the root causes for issues like the link between smoking and cancer or CKD-CTN (Table 4) took years. RCTs could not be conducted in these examples, as exposing volunteers to harmful factors would be unfeasible and unethical.

RCTs are not always necessary or practical for identifying causative factors in environmental or health issues (Table 3), such as hypovitaminosis D, acute life-threatening conditions, and behavioral disorders. Observational, prospective, and retrospective studies are often more cost-effective, easier to design [289], and provide outcomes closer to causality [284,289]. Determining root causes for issues like the link between smoking and cancer [267,268] or CKD-CTN (Table 4) has taken years [236,277,278]. However, RCTs are not feasible and could not have been ethically conducted in these scenarios, and subjecting volunteers to harmful exposures would be unfeasible and unethical.

### 6.5. Applying Hill’s Criteria for Vitamin D Deficiency as a Major Risk Factor for SARS-CoV-2

There is a robust association between hypovitaminosis D and the risk of contracting SARS-CoV-2 infection [59,74,131,146,147,170], as well as with the severity of COVID-19 clinical manifestations [237,244,257,259,260]. Multiple research groups have consistently observed this inverse relationship across various global locations [129,131,132,144,145,146]. This association remains significant even after adjusting for other relevant risk factors [64,74,131]. Furthermore, pre-pandemic hypovitaminosis D increases susceptibility and vulnerability to these risks [242,258,290,291]. Thus, it is unlikely due to reverse causality, affirming that vitamin D deficiency contributes to heightened susceptibility to this illness.

There is compelling evidence that low pre-infection 25(OH)D levels or at the time of hospital admission were also associated with increased vulnerability to contracting SARS-CoV-2 [60,116,117,118]—reported among unvaccinated Caucasians [292]. When reverse causality is genuine, it significantly lowers serum 25(OH)D concentrations [293]. Moreover, recent trial sequential analyses and meta-analyses further support vitamin D’s protective role against hospitalizations, especially in preventing ICU admissions [129]. Furthermore, there is a biological gradient—where more severe vitamin D deficiency is associated with increasing risks, severity, and mortality [62,64,65,129]. The known actions of calcitriol in immune cells provide biological plausibility [74,76,91,104,144,145,146,183,184,187]. Sufficiency of vitamin D is protective against severe disease and death [129,130,131,132].

### 6.6. Vitamin D Insufficiency Meets Bradford Hill Criteria for SARS-CoV-2 Susceptibility—Clinical Implications

To substantiate Bradford Hill’s criteria [148], well-designed clinical trials and observational studies should involve the supplementation of individuals with biochemically demonstrated vitamin D deficiency. Such participants are randomized to active treatment and control or a placebo group [190]. This approach has validated Hill’s criteria for vitamin D deficiency causing cancer [161,245,246] and multiple sclerosis [250,251]. The crucial mechanisms of action of intracellular calcitriol in immune cells further support the biological plausibility [64,91,142,161,188,257,258].

Published research data (over 300 clinical trials) suggest that vitamin D significantly reduces complications and deaths from SARS-CoV-2 [35,42,43,44,134,135,154,155,156,157,158,159,160,161,200,201]. Therefore, appropriate supplementation to avoid vitamin D insufficiency/ deficiency, with a proper control group receiving a placebo, is becoming ethically impossible and perhaps unnecessary. The SR data presented here establish that hypovitaminosis D fulfills Bradford Hill’s criteria for causality in SARS-CoV-2 infection and other viral illnesses. The findings are reproducible and reveal strong associations between severe vitamin D deficiency and heightened susceptibility to viral infections, along with an increased risk of complications [244,259,260] and fatalities from SARS-CoV-2 [21,90,154,158,207,208,209,210,211,212,262]. The key points from this systematic review are summarized in Table 5 below:

The above-mentioned findings provided a strong evidence-based rationale for recommending vitamin D_3_ and calcitriol as preventative and adjunct therapy against SARS-CoV-2 [21,56]. Despite this, health authorities and scientific organizations have failed to recommend (even to date) incorporating vitamin D_3_ as a preventive measure and D_3_ and calcifediol for early treatment (following exposure or first symptoms) in clinical practice guidelines and trials.

The data show that addressing vitamin D deficiency reduces disease vulnerability, symptomatic infections, and associated complications [244,259,260] and deaths from SARS-CoV-2 significantly [31,58,59,60,61,62,63,64,65,66,67,68,69,70,71,72,73,74,75,76,129,142,143,144,145,146,147,188,189]. Dismissing this evidence by claiming “there is no convincing evidence that vitamin D helps control SARS-CoV-2” is unscientific, unethical, and counterproductive [21,23,56], which harms the population. It is time for policymakers, scientists, and practitioners to recognize the substantial evidence supporting vitamin D’s role in mitigating disease risks.

Recent reviews and recommendations confirmed that those mentioned in this study taken over a longer duration would improve immune resilience, autoimmune disorders, and overall health [19,98,294]. In addition to musculoskeletal and immune functions, vitamin sufficiency improves maternal and children’s health, mental health, and brain functions and prevents cardiovascular disorders and cancer [27,32].

## 7. Discussion

This systematic review confirmed that vitamin D meets Hill’s criteria for causality, linking hypovitaminosis D as a cause that increases susceptibility to SARS-CoV-2, its complications, and mortality. The published evidence strongly supports low 25(OH)D levels as a significant risk factor for cardiovascular diseases, cancer, and infections, fulfilling Hill’s causality criteria. Hill’s criteria offer a valuable framework for investigating risk factors (Table 2), mainly using well-designed observational clinical studies (not relying solely on RCTs).

Randomized treatment trials are often impractical or unethical in the context of many micronutrient deficiencies (e.g., vitamins and trace minerals), certain health conditions (e.g., smoking and alcohol consumption), and specific public safety measures (e.g., wearing seat belts in vehicles and helmets while cycling). Similarly, no RCTs are feasible or performed using vitamin D in treating rickets. As shown in Table 4, numerous examples underscore the limitations of applying RCTs in these scenarios. This issue becomes particularly significant during crises (e.g., epidemics and pandemics), where conducting blinded studies with neutroceticals is neither practical nor ethical. Nutrient-based clinical trials should not rely on RCTs—they are designed to obtain regulatory approvals for pharmaceutical agents or medical devices.

Table 5 outlines Hill’s criteria, illustrating the associations between hypovitaminosis D and increased vulnerability to SARS-CoV-2, including complications, hospitalizations, and mortality. The synthesis of data from myriad studies supports the conclusion that hypovitaminosis D is not merely associated with but plays a causal role in increasing the risk and severity of SARS-CoV-2 infections. This conclusion aligns with Bradford Hill’s criteria, and vitamin D deficiency is a key biological determinant for increased vulnerability to SARS-CoV-2, derived from a weakened immune system. As illustrated in Table 2, these supporting factors establish causality in epidemiological research.

Making the above conclusions could have been expedited if a Big Data meta-analysis had been conducted in late 2020 on data from over 390,000 subjects available at that time [21,56]. It would have provided validation and affirmative evidence that hypovitaminosis D is causative for SARS-CoV-2 infection-related complications and deaths, enabling the recommendation of vitamin D as a preventative and adjunct treatment. This approach could have significantly reduced hospitalizations and saved lives. Although not all the stipulated criteria for causality suggested by Bradford Hill need to be satisfied, hypovitaminosis D satisfies all the criteria for the causation of increased risks of COVID-19—increasing vulnerability to the infection and markedly raising the risks of complications and deaths from COVID-19 illness. So, calling for additional studies and RCTs is unwarranted.

## 8. Conclusions

This SR confirmed that extensive evidence demonstrates that low vitamin D levels significantly increase the incidence, severity, complications, and mortality of various infections, including tuberculosis and viral respiratory illnesses in children [137] and adults [133,134], and especially for SARS-CoV-2 infection [69,70,141,171,241,242,243]. In addition, pre-existing vitamin D deficiency increases the risks of SARS-CoV-2 infection [60,74,147], its complications [71,73,147], hospitalizations [65,72,144,145,146], and deaths [71,129,132,142,143]. Administering the correct vitamin D supplements at the right frequency in deficient individuals significantly reduces the risks of infections, complications, and deaths from SARS-CoV-2 [60,65,71,72,73,74,129,142,143,144,145,146,147]. Many such studies provided strong evidence for causation.

Hypovitaminosis D significantly impairs the immune system, increasing vulnerability to infections, including SARS-CoV-2. Maintaining adequate vitamin D levels (e.g., above 50 ng/mL) enhances immune resilience, lowering the risk of symptomatic COVID-19 and reducing complications and deaths. Published evidence confirms that maintaining serum 25(OH)D concentrations in individuals above 50 ng/mL (40 ng/mL in the population) decreases symptomatic SARS-CoV-2 infections, disease severity, and deaths by more than 50%.

Data established vitamin D as a crucial preventative and adjunctive measure for mitigating COVID-19 risks in the population. Implementing public health initiatives to address widespread vitamin D deficiency in communities would substantially reduce the prevalence of acute infections, chronic diseases, hospitalizations, and premature mortality [22,27,32,98] and be highly cost-effective. Current guidelines are outdated and focus solely on bone health. These ineffective recommendations must be updated or replaced to reflect the extensive health benefits of vitamin D. Such revisions would enhance public health, decrease absenteeism, improve productivity, and significantly lower global healthcare costs.

## Figures and Tables

**Table 1:** PICOS Criteria—Participants; intervention; comparison; outcome elements; and study design philosophies.

	PICOS Criteria	Conditions
1	**Participants**	Adults aged 18 to 75 (males and females) infected with SARS-CoV-2; clinical trials. Including RCTs using vitamin D_3_ (cholecalciferol) and/or calcifediol (25(OH)D) as an intervention for those with SARS-CoV-2 infection.
2	**Intervention**	Observational and retrospective clinical studies or interventions and RCTs using vitamin D or calcifediol—focusing on vulnerability to infection and disease severity (hospitalization intensive care unit (ICU) admission) and deaths.
3	**Comparison/control**	Observational, community-based/ecological, RCTs, and meta-analyses were investigated with and without providing vitamin D or calcifediol, including outcomes from control groups.
4	**Outcome elements**	Focused on hard endpoints—morbidity, complications, comorbidities, hospitalization and ICU admissions, death, and all-cause mortality.
5	**Study design** **philosophies**	RCTs, non-randomized controlled clinical trials, non-randomized non-controlled trials, and prospective and observational studies, including ecological studies that have used vitamin D or calcifediol as an intervention prior to or after the diagnosis of SARS-CoV-2 infection.

**Table 2:** Bradford Hill Criteria: Vitamin D deficiency and its role in COVID-19 vulnerability, complications, and mortality [148].

**Criteria**	**Evidence**	**Evaluation and Explanation**
Strength of the association	Based on RCTs, meta-analysis, and cohort studies, hypovitaminosis D is associated with increased vulnerability to SARS-CoV-2 infection. The lower the serum 25(OH)D concentrations, the higher the severity and rate of deaths.	The stronger an association, the more likely it contributes to disease causality. While COVID-19 stems from a single pathogen, susceptibility increases with factors like obesity, diabetes, dark skin, overcrowding, and air pollution—factors known to reduce vitamin D levels. Adjusting for these causation analyses is complex due to their multifactorial nature. Despite the robust inverse associations of COVID-19 risks with pre-pandemic and pre-infection vitamin D status, these data were overlooked by regulators and health authorities [60,116,117,118,169]. In COVID-19, when vitamin D was used prophylactically [170], early in the treatment [129,131,132,144,145,146], or when patients treated with calcifediol [141,168,171], most RCTs reported greater efficacy with a large effect size associated with significant *p*-values.
Consistency	Properly conducted observational evidence from cross-sectional and longitudinal studies and RCTs confirmed that hypovitaminosis D increased the risks and complications from SARS-CoV-2. Vitamin D status is a biological determinant of immunity.	The available evidence from the pre-COVID era and prospective and retrospective clinical studies of vitamin D status concerning SARS-CoV-2 risks in different situations in multiple ethnic groups provides similar associations and outcomes in different locations. It has continued to do so over time. Inverse vitamin D status–SARS-CoV-2 relationships have been found repeatedly in multiple circumstances (community, outpatients, and in-hospital) [131,141,168,170,171] by many research groups in many different countries [129,169], which provides additional evidence for the consistency of the association [129,132,144,145,146], and also, in the pre-COVID [120,133,134,169,172,173] and post-COVID era [60,65,71,72,73,74,129,132,142,143,144,145,147].
Specificity	Hypovitaminosis D is prevalent among vulnerable populations, such as in nursing homes, disability centers, and dark-skinned people in temperate climates, leading to high death rates in these groups.	Does the experimental evidence point to a specific agent, location, or disease for the outcome? Differential exposure may give rise to a single result in certain situations [169,174], such as CKD arising from diabetes, hypertension, or exposure to toxins. However, this is not relevant to a single-cause disease like SARS-CoV-2. As discussed above, several factors increase susceptibility to viral illness. Aging and comorbidities increase the prevalence of hypovitaminosis D, which is the critical factor increasing COVID-19 risks or susceptibility to developing complications and deaths across all the groups studied. Chronic diseases like CVD and CKD often have multiple causes, including multigene factors, as seen in obesity and diabetes, leading to varied clinical outcomes. These diseases may lack one-to-one causality. In contrast, infections like COVID-19 primarily result from a single cause—the SARS-CoV-2 virus [62,129,131,144,145,146,170].
Temporality	Hypovitaminosis D precedes the onset of SARS-CoV-2 infections in the studied cohort.	Temporality is an essential criterion for establishing a causal association between exposure and outcome [29,73,127,142,175,176,177,178,179,180,181,182]—i.e., the exposure (SARS-CoV-2) must precede the outcome (COVID-19) [62,131,169,170]. Evidence suggests that clinical outcomes often remain unaffected by the intake of a single nutrient, like vitamin D, or the evaluation of hard outcomes. Chronic conditions such as cancer, CKD, or chemotherapy-induced appetite loss can independently influence outcomes. The overall nutrient status changes are more likely to affect these processes than the “experimental” nutrient, potentially worsening outcomes. Distinguishing cause–effect relationships from reverse causation is possible in some cases. Unlike severe bacterial pneumonia and septicemia, any slight reduction in serum 25(OH)D levels before COVID-19 hospitalization is clinically insignificant [59,74,144,145,146,147], not affecting Hill’s criteria. Pre-infection serum 25(OH)D levels are preferable [60,74,116,117,118]; studies recording vitamin D status pre-pandemic or pre-illness are the most relevant [74,120].
Biological gradient	Increased 25(OH)D concentrations are linked to better clinical outcomes. As a threshold nutrient, vitamin D intake does not have a linear relationship with serum 25(OH)D levels, making it unreliable to estimate serum levels based on oral dose. Effective serum 25(OH)D levels are 40–80 ng/mL without adverse effects.	This requires a demonstration of the dose–response association—curve: the greater the severity of the causal factor (the lower the serum 25(OH)D concentrations), the higher the risk of adverse health effects and outcomes (hospitalizations, complications, and death). Similarly, longer exposure and/or greater accumulation of a toxic agent (e.g., the SARS-CoV-2 viral load) increase the harmful effect. Moreover, when a condition is in its early stages, before irreversible structural damage occurs, eliminating the exposure should reduce adverse outcomes [62,131,141,168,170,171]. While the dose–response relationship of orally supplemented vitamin D to serum 25(OH)D concentrations achieved is not linear, a robust inverse relationship exists between them—i.e., serum 25(OH)D concentrations are higher with higher oral intakes of vitamin D, which protect from infections [169,183,184]. The indicated therapeutic levels of above 50 ng/mL also prevent symptomatic disease, complications, and deaths from SARS-CoV-2 [59,74,129,132,144,145,146,147]. The dose–response relationship between oral vitamin D intake and serum levels is complex, with the response influenced more by the degree of deficiency and body weight (including fat and muscle mass) than by the administered dose. Therefore, clinical outcomes should be correlated with the achieved serum levels rather than the oral dose.
Plausibility (mechanisms)	Vitamin D participates in the biology and physiology of the immune system. Thus, it is unsurprising that vitamin D sufficiency leads to a robust immune system and infection protection.	Probability or likelihood assumptions rely on prior beliefs, reports, or expectations rather than logic or data [185]. Developing plausible explanations is easier than empirically evaluating them [120,169]. For known mechanisms, such as cathelicidin and defensins in infections (which could also serve as biomarkers of severity or responses), their concentrations increase with increasing serum 25(OH)D concentration D, which enhances both innate and acquired immunity, e.g., preventing cytokine storms and reducing the risk of acute respiratory distress syndrome [60,65,71,72,73,74,76,129,142,143,147,186,187]. A multitude of published data related to vitamin D confirmed that low vitamin D status [i.e., circulating 25(OH)D cocentrations] pre-infection or at the time of hospitalization increases the risk of infection [60,74,116,117,118,134,183,185,188,189], similar to contracting COVID-19 as well [129,144,145,146,186,187]. Post-COVID syndrome, a chronic process, is initiated mainly in those with severe hypovitaminosis D [190], followed by the dissemination of infection into the central nervous system [191]. This prolonged infectious process continues, which consumes 25(OH)D, keeping it even lower (this is compatible with a reverse causality but is a separate entity).
Coherence (being logical and consistent)	A clear relationship between the two variables. Robust evidence that serum 25(OH)D levels are a key biological determinant that increases the vulnerability to viral infections and deaths, especially SARS-CoV-2.	Coherence and biological plausibility share typical constraints [76,187]. When evaluating an association, cause-and-effect interpretations must align with known facts of the disease’s natural history and biology [74,120,131,169,185]. This requires examining exposure patterns and biological effects of the observed disease patterns and outcomes. Trials, sequential analyses, and meta-analyses show that proper vitamin D supplementation during illness significantly reduces risks, including hospitalization [132] and ICU admissions [129,144,145,146]. In addition, in vitro and ex vivo data from Chausse et al. [104] and Xu et al. [192], pulmonary lymphocytes from patients with COVID-19 [193], and animal studies further support the role of vitamin D in activating T-cell immunity by intracellular calcitriol (i.e., worse pulmonary inflammatory in response to the intratracheal challenges of lipopolysaccharide) in vitamin D deficiency.
Experiments	Vitamin D supplementation reduces symptomatic disease incidence, complications, and mortality.	Empirical data: Examined whether preventive actions based on a demonstrated “cause-and-effect” association would modify the expected health outcomes (Koch’s postulates). Would the experimental data strongly support causal relationships with a larger effect size? The overall answer is yes, regarding vitamin D [60,65,71,72,73,74,129,142,143,147,186]. Unlike ecological (observational/epidemiological) studies, well-designed experiments (laboratory or clinical trials/RCTs) control variables and confounders or modify exposure. The data’s value depends on the study design and conduct. Multiple meta-analyses and trial sequential analyses confirm this relationship [73,129,142,177,178,179,180,181,182,194].

**Table 3:** Limitations of single bolus dosing and delayed interventions in addressing hypovitaminosis D and SARS-CoV-2, with comparators of early therapy.

Trial Authors/Year	Faulty Study Design (Example)	Reference
**Using a one-time, high dose of vitamin D**	**One-dose, oral administration of vitamin D, “late” in the disease**	
Murai et al., 2021	A single large bolus dose of 200,000 IU (oral) vitamin D in moderately ill hospitalized patients.	[34]
Guven et al., 2021	A single 300,000 IU bolus dose (IM) of vitamin D in critically ill late-stage patients in the ICU.	[214]
Juan et al., 2022	A single 140,000 IU bolus dose (oral) of vitamin D in males, age > 65, critical patients in the ICU.	[35]
Zangeneh et al., 2022	Severely ill, late-stage COVID-19 patients (ICU; *n* = 193) with a single bolus of 100,000 IU of D_3_ showed no benefit from vitamin D.	[36]
Cannata-Andia et al., 2022	A single dose of 100,000 IU D_3_ administered to severely ill, late-stage COVID-19 patients (*n* = 274) failed to improve progress or ICU admissions (COVID-VIT-D).	[37]
Mariano et al., 2022	A single oral dose of 500,000 IU was compared to a placebo (*n* = 115) in patients with mild to moderate illness. They reported no difference in mortality and progression of the disease.	[39]
Cervero et al., 2022	Compared to 10,000 vs. 2000 IU: the higher dose was marginally better.	[215]
Fairfield et al., 2022	Vitamin D treatment was associated with greater odds of extended hospitalization and mechanical ventilation—the retrospective, unbalanced study used small doses of OTC vitamin D (unquantified). Besides, participants in the vitamin-D-treated group were older and had more comorbidities and higher BMI.	[216]
Ullah et al., 2021	A cross-sectional uncontrolled study showed no benefit. No improvement in mortality.	[217]
Al Sulaiman et al., 2023	Moderate to severely ill patients (*n* = 177) had unknown amounts of vitamin D compared to unmatched participants (random: *n* = 288) who did not receive vitamin D. No information was provided on the dose of vitamin D, and the study was not standardized. There was no difference in ICU admissions, ventilation, or mortality.	[40]
**Comparator Trials:**	**Similar study designs, but vitamin D was administered “early” in the disease.**	
Annweilier et al., 2022	Compared 400,000 vs. 50,000 IU single dose administered early: significant improvement in mortality.	[38]
Zhong et al., 2023	Meta-analysis: single high doses (100,000 IU), analysis of five clinical trials (Murai; Cerero; Mariani; Rasogi; Annweilwer).	[232]
Rastgoli et al., 2022	Early therapy with 60,000 IU daily for 7 days that maintained serum 25(OH)D above 50 ng/mL for a few weeks showed positive outcomes in patients with mild to moderate illness.	[218]

**Table 4:** Examples of root cause identification leading to disease prevention and public safety.

Elements That Needed Root-Cause Identification	The Method Used to Identify the Root Cause	Reference
Tobacco smoking and lung cancer	The causality that smoking causes lung cancer was established through epidemiological and observational studies.	[267,268]
Identification of the source of cholera by Dr. John Snow	Dr. Snow used basic ecological approaches to identify the source of cholera outbreaks	[269,270]
Tuberculosis—identifications of clusters helping control the disease	Contact tracing has been pivotal in understanding and controlling the disease and identifying index cases.	[271,272]
Use of helmets to prevent head injuries	Demonstrated effectiveness in reducing head injuries and fatalities among cyclists and motorcyclists.	[273,274]
Seat-belt use in vehicles, saving lives	Proven to reduce significant injuries and deaths through observational studies.	[275,276]
Chronic kidney disease of unknown etiology (CKDu, now called CKD–crystal tubular nephropathy; CKD-CTN)	The cause was identified through field observations, followed by laboratory water testing for common ions and electron microscopic studies.	[236,277,278]
Observations revealed that vitamin B, C, D, B_12_, and folic acid deficiencies caused specific nutritional disorders like scurvy and beriberi	Careful observations and documentation led to the identification of several specific nutritional disorders. For example, scurvy due to vitamin C deficiency among submarine staff led to identifying root causes such as deficiencies in vitamin C.	[279,280,281,282]

**Table 5:** Summary of data from 300+ clinical trials applying Hill’s criteria to link hypovitaminosis D with SARS-CoV-2 risk and outcomes.

Criteria	Supporting Statistical Correlations and Clinical Outcomes
Consistency	Multiple studies across different populations and locations consistently show an inverse association between vitamin D levels and the risk and severity of SARS-CoV-2 infections.
Strength of association	Strong statistical associations are observed, with significant differences in infection rates and clinical outcomes between vitamin-D-deficient and -sufficient individuals.
Temporality	Evidence indicates that low vitamin D levels precede the onset of infection, establishing a temporal relationship necessary for causality.
Biological gradient	There is a precise dose–response relationship where lower levels of vitamin D correlate with higher risks and severity of infections, supporting the causality.
Plausibility	Biological mechanisms explain how vitamin D modulates immune responses, reducing the risk of infection and severity through effects on immune cell function and inflammation control.
Coherence	The association fits well with the current knowledge of vitamin D’s role in immune function, supporting a coherent narrative that aligns with known biological processes.
Experimental evidence	Intervention studies show that correcting vitamin D deficiency can improve clinical outcomes in viral infections, including SARS-CoV-2.

## Data Availability

Data included in the article are referenced in the article.
